# T84 air‐liquid interface cultures enable isolation of human bocavirus

**DOI:** 10.1111/irv.12567

**Published:** 2018-05-08

**Authors:** Verena Schildgen, Ylenia Longo, Monika Pieper, Oliver Schildgen

**Affiliations:** ^1^ Kliniken der Stadt Köln gGmbH Klinikum der Privaten Universität Witten/Herdecke Institut für Pathologie Köln Germany

Despite being frequently detected as a copathogen in respiratory infections, the human bocavirus has also been shown to be a major player in viral respiratory mono‐infections in all age‐groups.^1^, ^2^, ^3^, ^4^ However, although known since more than a decade, cultivation of the virus remains a major challenge and was so far limited to some selected laboratories, which made either use of primary air‐liquid interface cell cultures or not commercially available CuFi‐8 cells.^5^, ^6^

Recently, Ghietto and coworkers proposed that CaCo‐2 cells, a cell line derived from a colorectal tumor, could be used to study parts of the HBoV replication cycle, as these cells could be infected with HBoV and shed the virus.^7^ Without doubts, this is a major progress, as CaCo‐2 cells are easily available and do not require extensive and expensive cell culturing efforts. Nevertheless, especially with respect to the pathogenesis of HBoV strains causing respiratory infections, the CaCo‐2 model remains limited, although the study by Ghietto and coworkers unintendedly confirmed the previous assumption that HBoV displays a tropism to colorectal tumors.^8^, ^9^

In search for alternative cell culture models, we became aware of the cell line T84 (ATCC^®^ CCL‐248™), which originates from a colon metastasis of a bronchial tumor; the colorectal localization of the primary tumor from which T84 derived as well as the fact that the tumor was of bronchial origin made the cells thus an ideal candidate cell line to test, as two putative perquisites for HBoV growth; that is, tumor type(s) and target/organ tissue were fulfilled. T84 cells could be easily grown as monolayer cultures and by mounting them on filter membranes coated with rat or human collagen can be differentiated into a multilayer respiratory epithelium (detailed protocols on request). None of the culturing steps requires any specialized cell culture media, thus making the cell line an economically acceptable tool for research.

To test whether T84 air‐liquid interface cultures are permissive for HBoV, we have infected the air surface of the cells with different volumes (5 μL, 10 μL, 50 μL, and 100 μL) of a virus stock of HBoV (3.9 × 10^10^ geq/mL). After 3‐4 weeks of culture in on filter membranes, the polarity of the T84 cells was determined based on the transepithelial electrical resistance (TEER) using an epithelial volt‐ohm meter (Millipore). HBoV1 infection was performed at minimal values of 600‐800 Ω per insert, which most likely goes ahead with differentiation of the cells. Six hours after inoculation, the cells were washed three times before the first basal medium was harvested 24 hours post‐inoculation. HBoV infectious particles were thereby derived from HEK293 cells transfected with an HBoV‐1 plasmid as previously described by Huang et al,^5^ 2014.

**Figure 1:**
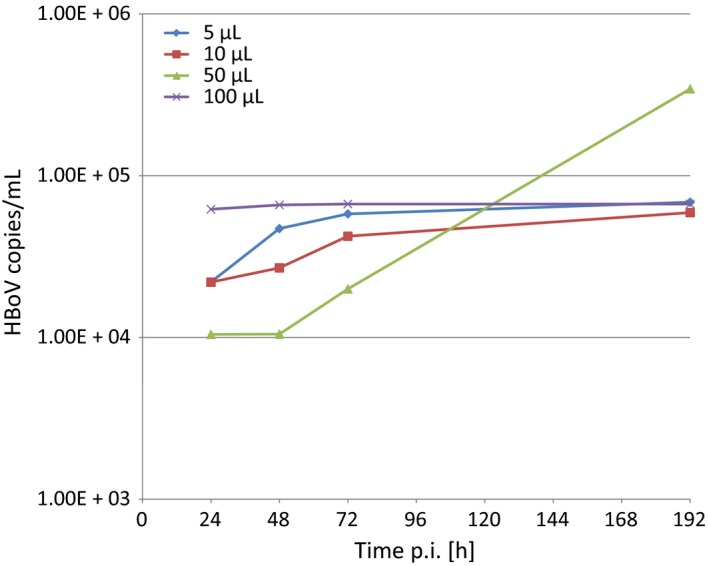
Representative growth kinetics of HBoV in basal media of T84 air‐liquid interface cell cultures

As an active HBoV infection is characterized by a viremia, that is, a release of viral particles into the blood stream, HBoV replication was measured by the number of HBoV DNA copies that were released to the basal cell culture medium. Except for 100 μL inoculum, we did observe an increase in viral particles up to one log10 in all cases (Figure 1), indicating that active replication occurred and viral particles were released to the basal cell culture medium. None of the filters became leaky, and even after 7 days of infection, no cell culture medium was visible on the apical surface of the air‐liquid interface cultures. In addition, we have tried to isolate HBoV from bronchoalveolar lavages. In 4 of 8 attempts, we were able to observe the release of viral DNA into the basal medium, giving raise to the assumption that the T84 cell culture system could also become a tool for classical virus isolation. Further preliminary approaches with productive infections support the conclusion that passaging of HBoV on T84 may also be possible as the medium of T84 cells freshly inoculated with medium previously tested positive also became positive by qPCR. Although this has to be confirmed by further trials, T84 could supplement the CaCo‐2 model and, in concert with the CaCo‐2 cell model, could serve as a tool for studying HBoV replication and identification of host factors that enable the cell type–specific mechanisms leading to productive HBoV infection. Moreover, as broadly available cell lines, CaCo‐2 and T84 could serve as a basis for more HBoV research even in less specialized laboratories.
